# Diphtheria

**Published:** 1889-05

**Authors:** Horace Still

**Affiliations:** Norristown, Pa.


### DIPHTHERIA.

Horace Still, M. D., Norristown, Pa.

This is defined to be a specific, contagious, asthenic disease which sometimes prevails as an epidemic, and is endemic in some localities.

It is characterized by the exudation of a false membrane on the mucous surface of the soft palate, uvula, tonsils, pharynx, larynx, and trachea, or it may involve the posterior nares and eustachian tube, or it may appear in more remote localities. Together with this false membrane there is more or less marked prostration of strength, the appearance of albumen in the urine, sometimes a cutaneous eruption, enlargement of glands, and where the larynx and trachea become involved, the distressing croupy symptoms appear. These, together with other symptoms known to all, constitute the picture of the disease. But, rather than a description of the disease, it is the treatment which is of the most interest to the homœopathic physician. Right here, let me say, it is in this disease that some of the greatest triumphs of Homœopathy have occurred, while, on the other hand, the mortality on the part of the old school is large, the treatment unsatisfactory, as is evidenced by the utter want of well-credited and successful measures, and their constant seeking after something which will destroy the so-ealled micrococcus diphtheriticus, and thus, as they mistakenly suppose, cure the disease. There is only one true way of curing an individual afflicted with symptoms which are termed diphtheritic, and that is by strict adherence to the homœopathic law of cure, founded upon its three (3) fundamental principles:

1. The Similar Remedy.
2. The Single Remedy.
3. The Minimum Dose.

There is no one specific for diphtheria, but every case must be carefully and patiently individualized.

REMEDIES AND THEIR INDICATIONS.

*Ailanthus g.*—Diphtheria with scarlatinal complications: where there is a livid and swollen throat and tonsils studded with numerous deep, angry-looking ulcers, exuding a scanty, fetid discharge; livid, purplish appearance of the skin; semi-conscious condition, or entire insensibility.

*Ammon. caust.*—Especially in diphtheritic croup: marked hoarseness; low, husky cough; suffocative spells with great anguish; breathing hurried; pulse rapid, feeble, wiry. The whole throat covered with a white exudate, with intense pain in throat; great difficulty in swallowing; great weakness and prostration, not in proportion to the short duration of the disease.

*Apis m.*—This remedy, as you well know, is so highly recommended by Jahr in his forty years' practice as to lead one to believe it to be a specific for nearly all cases; it does not, I think, occupy any such position in the homœopathic materia medica, but should be prescribed only according to well-defined indications.

There is marked debility from the beginning; throat presents a varnished appearance; the membrane is of a dirty gray color; and, although the parts are highly inflamed, there is comparatively little pain; uvula often becomes œdematous and looks as if filled with water; the margins and a little beyond the membrane are fiery red and shining, and this fiery margin moves as the membrane increases; pain in the ears when swallowing; often a stinging pain in the throat between the acts of deglutition; the throat, externally, is often swollen and puffy; thirstlessness; there is often a sensation of rapid swelling of lining membrane of throat; sense of suffocation, can bear nothing about the throat; skin perspires, and dries up in starts; urine scanty, albuminous.

*Arsen. alb.*—Sometimes called for with the characteristic restlessness, thirst, prostration, aggravation after midnight and albuminous urine.

*Arsen. jod.*—Recommended where the deposit extends even to the outer edge of the lips; foul breath; short, difficult respiration; marked adynamic symptoms; glandular enlargements.

*Arum tri.*—Mouth burns and is sore, so that they refuse to drink; discharge of a burning, ichorous fluid from nose, excoriating the upper lip; nose stopped up and they can only breathe with the mouth open, this is the case with or without discharge from the nose; picking at lips and nose, making them bleed; fetid breath; sensation of something hot in throat; hemorrhage from nose, mouth, and throat; great restlessness.

*Baptisia.*—Very little pain, fauces œdematously swollen, with constant inclination to swallow; membrane has a dark appearance; breath offensive; suffocating spells; can only swallow liquids; prostration; stools dark and blood streaked; mind wandering; low, muttering delirium.

*Bell.*—Useful sometimes in beginning; restlessness; desire to swallow, and sensation as if he would choke if he did not swallow; great difficulty in swallowing solids or fluids; throat highly congested, bright red; right sided aggravation; sleepy but cannot sleep; pupils dilated; red face, etc.

*Bromium.*—Croupous symptoms; recommended for diphtheria apparently commencing in larynx and spreading upward; stiff neck with diphtheria; hoarse, croupy cough.

*Cantharis.*—Burning in throat, with scraping sensation and spitting of blood; spasmodic constriction and intense pain at the back part of the throat; dysuria; urine albuminous and shreddy; extreme prostration; sinking, death-like turns; rash on skin or shining through.

*Capsic. an.*—Burning and soreness in mouth and throat; fauces covered with a considerable deposit, with smarting, beating, and throbbing in the head; rapid pulse; vertigo; epistaxis; chilliness in the back.

*Ignatia.*—This remedy, as you are aware, was first introduced in the treatment of diphtheria, by Dr. Boskowitz, of Brooklyn. It was subsequently used by Dr. W. C. Slough, in an epidemic in Lehigh County, this State, with marked success, and that, too, with the 200th trituration. The symptoms characterizing this epidemic were "green vomiting; putrid throat, seldom painful (the painful cases were less likely to prove fatal); greenish-yellow patches; delirium, headache; green stools; suppression of urine, sometimes chilliness, sometimes high fever."

This remedy, also, is most likely to be useful where the right side is affected, although the exudation may be on both sides; high fever with delirium, characterized by fearfulness or dread; soreness of throat, worse between the acts of deglutition; pain in the back of the head, trachea, and sometimes in the ears.

*Aggravation.*—When not swallowing (between the acts), and when swallowing liquids.

*Amelioration.*—When swallowing food.

*Kali bich.*—Tough, stringy discharge from the nose, which is often fetid; pharynx red, swollen; thick; tenacious, ashy-gray membrane, which has a strong tendency to spread downward to larynx; smell from the mouth as of decayed meat; uvula looks like a bladder; parotid glands swollen; harsh and stridulous breathing; voice partially suppressed; an almost unconscious condition when aroused from it or when awaking from apparent sleep; marked aggravation; awakens with desire to cough or to hawk up detached portions of the diphtheritic deposit, and throws off tough, ropy, yellow mucous expectoration, frequently streaked with blood; extreme prostration.

*Kali permang.*—Fauces covered with a peculiar wash-leather, grayish membrane, breath offensive from the beginning; thin, watery, sainous discharge from nose, excoriating the upper lip; fluids taken by the mouth return through the nose; vomiting; dark-colored, offensive diarrhœa; general prostration; sometimes a comatose state.

*Kali phos.*—Has been recommended where there is a marked putrid, gangrenous condition and a fearful stench from the mouth.

*Lac can.*—One side of the nose stopped up, the other free and discharging thin mucus at times and thin blood, these conditions alternate, first one nostril is stopped up and the other fluent, and *vice versa;* fluids escape through nose while drinking; on swallowing, acute pains at one time on right side of the throat, and at another on the left side; diphtheritic membrane, white like china; mucous membrane of the throat glistening as if varnished; membrane changes sides repeatedly; desires for warm drinks; there may be difficult breathing, suffocative spells; pulse weak and rapid; tongue dry and coated grayish-white. This remedy is often useful after Lachesis.

*Lach.*—Commences on left side and goes to the right; aggravation from empty swallowing, less from liquids, relief from solids. Fluids return through the nose; spasmodic constriction of throat worse after sleep or arousing from sleep; intolerance of anything touching the throat; fever marked; pulse weak and rapid; great restlessness and prostration; sometimes delirium, with great loquaciousness and changing from subject to subject; useful in diphtheritic croup.

*Lachnanthes t.*—Useful in cases with stiffness of neck and head drawn to one side during or after the attack.

*Lycop.*—Disease commences on the right side or beginning in the nose; nose stopped up; not able to breathe through the nose, so that they are obliged to keep the mouth open, with tongue partly projecting, producing a silly expression on the face; wring-like motion of alæ nasi on awaking out of a short sleep, patient is often cross or will jump up in bed and stare and not recognize anybody; aggravation from hot drinks, they make the throat smart.

*Merc. cy.*—Useful in putrid forms of diphtheria; likely to commence in nostrils and spread downward; grayish-leathery exudation; much salivation; great fetor; excessive prostration and danger of collapse from commencement.

*Merc. jod. flav.*—Worse on right side; thick, dirty-yellow coating at base of tongue, tenacious mucus in throat; offensive odor from mouth; glands swollen; aggravation from warm drinks.

*Merc. jod. rub.*—Worse on left side; swallowing both of fluids and solids painful; exudation limited, transparent, easily detached; gums and tongue swollen and sensitive.

*Naja tri.*—Patient grasps at throat with sensation of choking; must sit up; breath fetid; short, hoarse cough; raw feeling in the upper part of trachea; blue appearance of the skin; pulse intermittent, thready; threatening cardiac paralysis.

*Nit. ac.*—Membrane on fauces and tonsils extends to the nose; stoppage of nose or corroding discharge from the nose; terrible fetor; swollen parotids; pain as from a splinter in the throat, worse when swallowing; intermittent pulse.

*Phytolacca dec.*—Dirty, wash-leather membrane; mucus hawked with difficulty from posterior nares, from which it hangs down in strings; excessive fetor of breath at times; severe aching of head, back, and legs; great prostration, with faintness on rising.

*Sul. ac.*—Thick yellow membrane on fauces and tonsils, very tenacious; deglutition impeded; voice thick; swelling of parotids; marked fetor from mouth; much weakness; excessive paleness.

*Sulphur.*—Yellow deposit about posterior wall of pharynx; quick pulse; flashes of heat; faintness; sinking spells; complaining of closeness of room, in sluggish cases.

